# The Beneficial Effects of Fish Oil Following Cisplatin-Induced Oxidative and Histological Damage in Liver of Rats

**Published:** 2017

**Authors:** Osman Ciftci, Elif Onat, Aslı Cetin

**Affiliations:** a *Department of Pharmacology, Faculty of Medicine, University of Inonu, Malatya, Turkey.*; b *Malatya State Hospital, Home Health Service Unit, Malatya, Turkey.*; c *Department of Histology and Embryology, Faculty of Medicine, University of Inonu, Malatya, Turkey.*

**Keywords:** Cisplatin, Fish oil, Hepatotoxicity, Oxidative damage, Histological damage

## Abstract

This study investigated the protective effect of fish oil (FO) on cisplatin (CP) toxicity in the rat liver. Twenty-eight rats were divided equally into four groups, with the first being a control group. The second group (CP group) was given 7 mg/kg of CP and the third group (FO group) was given 1 FO softgel/rat/day for 14 days. The rats in the fourth group (CP + FO group) were treated with both CP and FO at the above doses. CP treatment caused significant oxidative damage via an increase in thiobarbituric acid reactive substances (TBARS) and reduced antioxidant defenses through a decrease in the activities of catalase (CAT), superoxide dismutase (SOD), reduced glutathione (GSH), and glutathione peroxidase (GPx) in rat liver tissue. Also, CP caused histopathological abnormalities, including necrosis, in the liver tissue. However, concurrent FO treatment prevented the negative oxidative and histopathological effects of CP. In conclusion, CP treatment can cause hepatotoxicity in rats, but dietary supplementation with FO can attenuate the oxidative and histological changes caused by CP. Thus, FO may be useful in preventing CP-induced hepatotoxicity in cancer patients.

## Introduction

Cisplatin (CP, Cis-diamminedichloroplatinum II) is an effective chemotherapeutic agent used in the treatment of multiple types of cancer (1). However, the usage of CP is limited because of its severe side effects, including ototoxicity, nephrotoxicity, and hepatotoxicity (2). At high doses, CP treatment frequently causes hepatotoxicity, but little is known about the mechanism of CP-induced hepatotoxicity. Several reports have implicated reactive oxygen species and oxidative stress in CP toxicity (3, 4). 

Multiple studies (5, 6 and 7) indicated that antioxidants and other natural compounds may protect against CP toxicity. In addition, specific antioxidants may protect against CP-induced hepatotoxicity, including lycopene (8), caffeic acid phenethyl ester (CAPE) (9), and thiamine pyrophosphate (TPP) (10). However, information about fish oil (FO) treatment and the reduction or prevention of CP-induced hepatotoxicity is limited.

FO is a rich dietary source of ω-3 polyunsaturated fatty acids such as eicosapentaenoic acid (EPA) and docosahexaenoic acid (DHA). Recent studies (1, 11 and 12) report that FO is beneficial and protective against various disorders such as cardiovascular disease, cancer, Alzheimer’s disease and obesity. El- Mesery (13) found that CP (5 mg/kg) treatment alone produced a 55% reduction in tumor size in mice. Interestingly, treatment with low- (125 mg/kg) and high-dose (250 mg/kg) DHA alone also decreased tumor size by 38% and 79%, respectively. Combining a low dose of DHA with CP decreased tumor size by 81%. Additionally, in the same study, it was demonstrated that DHA treatment ameliorated CP-induced nephrotoxicity via changes in malondialdehyde (MDA) and glutathione (GSH) levels. 

We hypothesized that FO would prevent CP-induced hepatotoxicity due to its intrinsic biochemical and antioxidant properties and investigated the oxidative and histopathological changes in the liver tissue of rats treated with CP and FO.

## Experimental

*Chemicals*

CP (10 mg/10 mL, code 1876A) was obtained from Faulding Pharmaceuticals PLC (Warwickshire, UK). FO was given from Solgar (Leonia, NJ, USA) and one softgel contained 1000 mg FO and other substrates (vitamin E from soy, 180 mg EPA, and 120 mg DHA). All other chemicals were purchased from Sigma Chemical Co. (St. Louis, MO) and were of analytical grade or the highest grade available.

*Animals*

Rats were randomly divided into four groups (n = 7 per group). CP was administered intraperitoneally (i.p.) as a single 7 mg/kg dose. FO was administered by gavage at a dose of 1 softgel/day for 14 consecutive days. The control group was given a single isotonic saline i.p. injection and corn oil orally for 14 days. The CP group was given as a single injection of CP i.p. and corn oil for 14 days. The FO group was treated with FO orally for 14 days. with no CP. The CP + FO group was treated with CP and FO as described above. Tissue samples were collected on day 14. 

Animals were euthanized under ether anesthesia and liver tissue was removed immediately and dissected on ice-cold glass. Tissue samples were stored at –86 °C until analysis.

**Table 1:** The levels of SOD, CAT, GPx, GSH and TBARS in rat liver tissue. (n = 7, means ± SD).

	**TBARS (nmol/g tissue)**	**GSH (nmol/mL)**	**SOD (U/mg protein)**	**CAT (k/mg protein)**	**GPx (U/mg protein)**
Control	7.00 ± 0.61^a^	198.0 ± 10.9^a^	6.45 ± 1.01^a^	0.126 ± 0.012^a^	5.01 ± 0.19^a^
CP	10.89 ± 1.30^b^	110.4 ± 16.5^b^	4.82 ± 0.90^b^	0.084 ± 0.015^b^	3.85 ± 0.13^b^
FO	5.69 ± 0.27^c^	243.8 ± 27.8^c^	6.34 ± 0.87^a^	0.137 ± 0.015^a^	4.95 ± 0.23^a^
CP + FO	6.84 ± 0.88^a^	188.5 ± 34.0^a^	5.05 ± 1.05^b^	0.104 ± 0.004^c^	4.08 ± 0.20^b^

The mean differences the values bearing different superscript letters within the same line are statistically significant. (*P* ≤ 0. 01) SD: standard deviation.

**Table 2:** Comparison of the effect of FO on microscopic damage caused by CP in liver.

	**Microscopic damage (Mean ± SE)**
Control	0.45 ± 0.72^a^
CP	2.49 ± 0.78^b^
FO	0.69 ± 0.88^a^
CP + FO	1.82 ± 0.86^c^

The mean differences the values bearing different superscript letters within the same line are statistically significant. (*p* ≤ 0. 01) SE: standard error.

**Figure 1:**
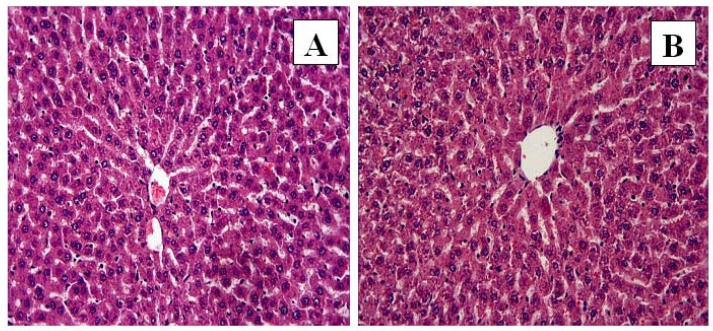
Control and FO groups: In control (A) and FO (B) groups, the liver showed normal histological appearance. H-E; X20

**Figure 2:**
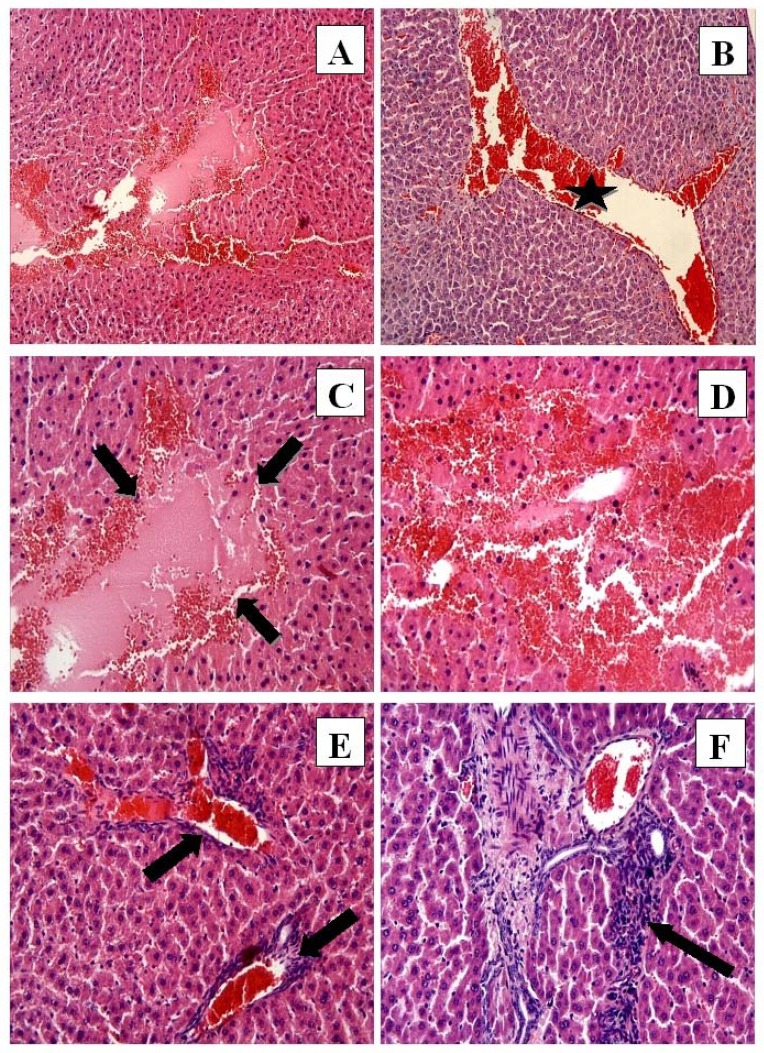
CP group: (A) Hemorrhage, eosinophilic stained pyknotic nuclei cells, necrosis H-E; 10; (B) Congestion (star) H-E; X10; (C) Necrosis (arrows) and hemorrhage H-E; X20; (D) Eosinophilic stained pyknotic nuclei cells and hemorrhage H-E; X20; (E) Congestion and mononuclear cell infiltration (arrows) H-E; X20; (F) Mononuclear cell infiltration. H-E; X20

**Figure 3:**
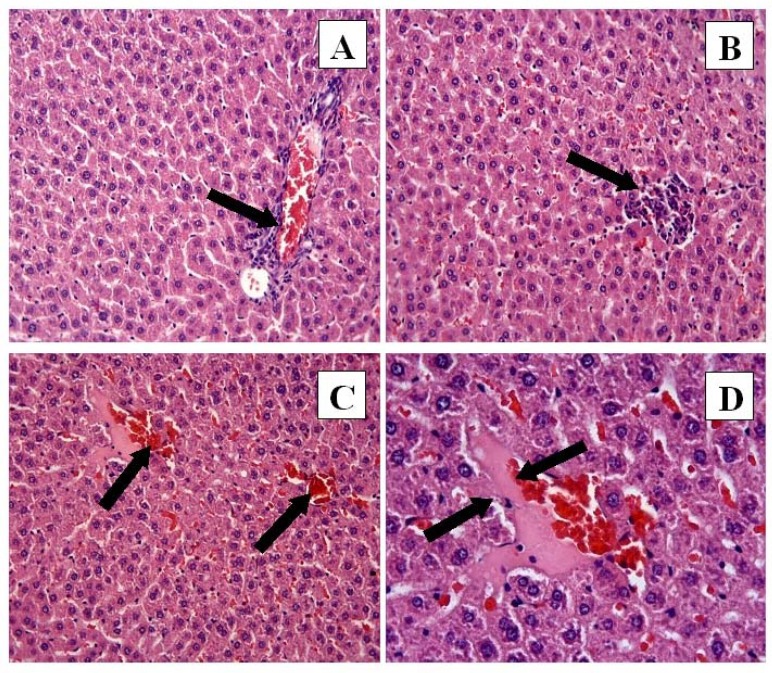
CP + FO group: (A) Mononuclear cell infiltration (arrow) and congestion. H-E; X20. (B) Mononuclear cell infiltrasyonu. H-E; X20. (C) Hemorrhage (arrows). H-E; X20. (D) Necrosis (arrows). H-E; X40

*Biochemical Analyses*

The homogenization of tissues was carried out in a glass Teflon homogenizer in a 150 mM KCl (pH 7.4) solution to obtain a 1:10 (w/v) dilution of the whole homogenate. The homogenates were then centrifuged at 18,000 g at 4 °C for 30 min to determine the levels of thiobarbituric acid reactive substances (TBARS), glutathione (GSH), and catalase (CAT). The homogenates were centrifuged at 25,000 g at 4 °C for 50 min to determine the levels of glutathione peroxidase (GPx) and CuZn-superoxide dismutase (SOD).

The level of TBARS in homogenized brain tissue was used as an index of lipid peroxidation and determined by the thiobarbituric acid reaction using the methods developed by Yagi (14). The homogenized solution was evaluated using a spectrophotometer at 532 nm, and the results were presented as nmol/g tissue. SOD activity was determined by the inhibition of the nitroblue tetrazolium (NBT) reduction by oxygen generated from the xanthine/xanthine oxidase system (15). One unit of SOD activity was defined as the amount of protein causing a 50% inhibition of the NBT reduction rate. The product was evaluated using a spectrophotometer at 560 nm, and the results were presented in IU/mg protein. CAT activity in the tissues was measured according to the method developed by Aebi (16). The enzymatic decomposition of hydrogen peroxide (H_2_O_2_) was followed directly by the decrease in absorbance at 240 nm. The difference in absorbance per unit of time was used as a measure of CAT activity, and the enzyme activities were presented in k/mg protein. GPx activity was determined according to the method developed by Paglia and Valentina (17). In the presence of GSH reductase and NADPH, the oxidized glutathione (GSSG) is immediately converted to its reduced form in conjunction with the oxidation of NADPH to NADP. The decrease in absorbance at 340 nm was measured, and GPx activity was presented in IU/mg protein. The GSH content of the homogenate was measured at 412 nm using the method developed by Sedlak and Lindsay (18) and GSH levels were presented in nmol/mL protein. Tissue protein content was measured according to the method of Lowry *et al.* (19) using bovine serum albumin as a standard.

*Histological evaluation*

For light microscopic evaluation, liver tissues were preserved in 10% buffered formaldehyde and embedded in paraffin wax. Paraffin-wax-embedded specimens were cut into 5-μm-thick sections, mounted on slides, and stained with hematoxylin and eosin. Tissue samples were examined using a Leica DFC 280 light microscope with a Leica Q Win Image Analysis system (Leica Micros Imaging Solutions Ltd., Cambridge, UK). 

The severity of liver damage was assessed semiquantitatively using the following criteria: hepatocytes with eosinophilic cytoplasm, hepatocytes with pyknotic nuclei, necrosis, hemorrhage, congestion, and mononuclear cell infiltration. Microscopic damage was scored as absent (0), slight (1), moderate (2), or severe (3), for each criterion.

## Results

*Biochemical results*

Levels of superoxide dismutase (SOD), catalase (CAT), glutathione peroxidase (GPx), GSH, and thiobarbituric acid reactive substances (TBARS) in liver tissue are given in Table 1. CP caused a significant increase in liver tissue TBARS levels. GSH, CAT, SOD, and GPx levels were decreased significantly in the CP group compared to the control and FO groups. However, FO treatment combined with CP caused a significant reduction in TBARS levels compared to CP alone, and the TBARS levels in the CP + FO group were lower than in the control group. Furthermore, in the CP + FO group, GSH and CAT levels were significantly elevated compared to the CP-alone group. In contrast, SOD and GPx levels were not significantly different between the CP + FO and CP groups.

*Histological results*

The liver tissue of the control (Figure 1A) and FO (Figure 1B) groups demonstrated normal histological structure. In the control and FO groups a normal radial arrangement of hepatocytes was observed, and sinusoids and central veins were also evident.

In the CP group the liver tissue had significant histological alterations consisting of necrosis (Figure 2A, C), hemorrhage (Figure 2A, C, D), eosinophilic staining and pyknotic nuclei cells (Figure 2C, D), congestion (Figure 2B, E), and mononuclear cell infiltration (Figure 2E, F). The normal radial arrangement of hepatocytes from the central vein was also disrupted.

However, the histological changes were decreased in the CP + FO group. Eosinophilic staining and pyknotic nuclei were observed in the CP + FO group, but they were not as widespread as in the CP group. The negative histopathological findings of the CP + FO group were not as extensive as the CP group (Figure 3A, B, C, D).

The microscopic damage score for each group is given in Table 2. CP treatment caused a significant increase in microscopic damage to the liver tissue, and FO treatment with CP caused a significant reduction in the microscopic damage observed with CP treatment alone.

## Discussion

CP treatment can cause many negative side effects in cancer patients, which can have a dramatic impact on quality of life. The prevention of CP side effects is important not only for patient quality of life but also because this could potentially increase dosage limits. In this study, CP-induced hepatotoxicity involved increased lipid peroxidation, decreased antioxidant status, and histopathological defects. Our findings also demonstrated that FO could prevent the hepatotoxic effects of CP when used in combination with CP. 

Oxidative stress is an imbalance between free radicals and the molecules of the antioxidant defense system, including SOD, CAT, GPx (enzymatic), and GSH (non-enzymatic). Under normal cellular conditions free radicals are immediately detoxified by the antioxidant defense system. However, excessive free radical production causes an imbalance, which leads to lipid peroxidation and antioxidant depletion (20). Little is known about the mechanism of CP-induced hepatotoxicity, but multiple studies have reported that CP causes significant oxidative damage. Lu and Cederbaum (21) reported that CP-induced hepatotoxicity is enhanced by an increase in the generation of reactive oxygen species and oxidative stress. Naqshbandi *et al.* (4) demonstrated that in rats CP treatment caused a 52% elevation in lipid peroxidation and reduced the activities of SOD, CAT and GPx by 15%, 70% and 76%, respectively. Also, several previous studies of liver oxidative status reported that CP treatment resulted in significant oxidative damage (9, 22). Additionally, numerous studies have reported that in addition to its effect on the liver, CP also induced oxidative stress damage in the kidney (23), testis (20), ovary (24), brain, and sciatic nerve (25). However, Al-Majed (3) asserted that oxidative stress is not the main cause of CP-related hepatotoxicity and indicated that the mechanism of CP-induced hepatotoxicity could be related to carnitine deficiency.

The results of the present study are consistent with the majority of previous reports, and indicated that CP treatment results in significant oxidative damage to liver tissue, and that CP-induced lipid peroxidation in liver tissue, increasing TBARS levels significantly. Also, SOD, CAT, GPx, and GSH levels were decreased by CP treatment. These results indicate that CP treatment leads to an imbalance in the oxidative system, which causes oxidative damage in the liver.

Previous studies have shown that FO has hepatoprotective properties and could be used to combat the oxidative effects of toxic agents such as CP. El-Mowafy *et al.* (26) demonstrated that EPA, which is found in FO, markedly alleviated valproate-induced hepatotoxicity. Similarly, Speck and Lauterburg (27) and Kuralay *et al.* (28) reported that FO prevented acetaminophen hepatotoxicity *in-vivo*,and Kuralay *et al.* (28) demonstrated increased GSH in the liver. Fallon *et al.* (29) reported that an FO-based emulsion exerted hepatoprotective effects in parenteral nutrition-associated liver disease. 

To our knowledge, only two studies of the protective effect of ω-3 fatty acids on CP-induced hepatotoxicity in rats have been published. Naqshbandi *et al.* investigated the protective effects of FO (1) and flaxseed oil (4), which also contains ω-3 fatty acids, in CP-treated rats. Both studies demonstrated that FO and flaxseed oil reduced lipid peroxidation and induced SOD, CAT and GPx activity compared to in CP-treated rats. These results are consistent with the findings of the present study, which demonstrate that FO administered with CP treatment can prevent oxidative stress, reduce TBARS levels and increase CAT and GSH levels. However, in the present study SOD and GPx levels did not differ significantly in the CP + FO group compared to the CP group.

The histopathological evaluation demonstrated that CP treatment produced histopathological alterations in the liver tissue, including necrosis, hemorrhage, increased eosinophilic staining, pyknotic nuclei cells, congestion, and mononuclear cell infiltration. These data were supported by the microscopic damage scores. FO treatment alone produced no histopathological change in liver tissue when compared to controls. However, histopathological damage decreased when FO was given in combination with CP. Multiple previous studies (22, 30) demonstrated that CP treatment increased histopathological damage in liver tissue, and that antioxidant treatment protected liver tissue from this damage, which is consistent with our findings. It is possible that the histopathological effects of CP treatment are due to the CP-induced imbalance between free radicals and the antioxidant defense system. Additionally, this imbalance may contribute to the development of severe hepatic disorders, such as liver failure. Therefore, decreasing oxidative stress in the liver by FO treatment will combat the effects of CP-induced hepatotoxicity and prevent the development of hepatic disorders.

## Conclusions

CP treatment induced oxidative and histopathological changes in rat liver tissue. FO in combination with CP treatment generally prevented CP-induced hepatotoxicity. The beneficial effects of FO are likely due to its polyunsaturated fatty acid, such ω -3 fatty acids, components, and their antioxidant and anti-inflammatory properties. Therefore, ω-3 fatty acid-enriched FO can protect against CP-induced hepatotoxicity and dietary supplementation with FO may benefit cancer patients being treated with CP. 
